# Exploring long noncoding RNAs as a molecular connection between immune response and autism spectrum disorder

**DOI:** 10.3389/ftox.2026.1893084

**Published:** 2026-10-16

**Authors:** Anaya Roschyk, Daniel B. Campbell

**Affiliations:** College of Human Medicine, Michigan State University, East Lansing, MI, United States

**Keywords:** autism, autism spectrum disorder, brain, immune, lncRNA, long noncoding RNA

## Abstract

Autism spectrum disorder (ASD) is a neurodevelopmental condition increasingly linked to immune dysregulation, with long noncoding RNAs (lncRNAs) emerging as potential regulators of both neural and immune processes. This study commenced by analyzing ten unique lncRNAs and integrated ASD whole-blood datasets using the online platform GTex to identify expression of lncRNAs in various tissues. Moreover, the platform Network Analyst was utilized to assess differentially expressed lncRNAs and associated transcriptomic networks. A total of 93 upregulated and 51 downregulated lncRNAs were identified in people with ASD with tissue specific enrichment observed for *MSNP1AS* and *LNC01238* in immune related systems. The transcription factor CEBPA was shown to interact with the upregulated lncRNA *MALAT1*, which influenced immune cell differentiation, inflammation and cytokine signaling. Intriguingly CEBPA simultaneously interacted with downregulated lnc RNA *MIR22HG.* Comparatively, downregulated lncRNAs were associated with the transcription factors TEAD2, CEBPA, CTCF, E2F4, TFAP2C, HIF1A, ETS1, HNF4A, SNAPC4, STAT1, STAT2, TEAD2, FOXC1, BRCA1, AR, E2F4, EOMES, SPI1, DACH1, E2F1, TP63, HMAP1, KLF9, HDGF, and MAZ. Overall, the findings support a role for lncRNAs in ASD pathophysiology and immune dysregulation, pointing to their potential as biomarkers and potential therapeutic targets for future research.

## Introduction

Genetics can provide important insights into the molecular pathways that are impacted in neurodevelopmental disorders (Zhou et al., 2019; Xia et al., 2015; Xia et al., 2014; Levitt and Campbell, 2009; Baron et al., 2006a; Baron et al., 2006b). Substantial exome sequencing efforts identified *de novo* loss-of-function mutations that contribute to autism spectrum disorder (ASD) risk (An et al., 2018; O’Roak et al., 2012a; Neale et al., 2012; Sanders et al., 2012; Iossifov et al., 2012; O’Roak et al., 2012b; Bernier et al., 2014; An et al., 2020; Ayhan and Konopka, 2019; Satterstrom et al., 2020). These mutations provided important insights into the biological pathways that contribute to ASD risk. Whole genome sequencing of individuals with ASD confirmed that ∼10% of ASD cases can be attributed to protein-coding genes, and has shown that noncoding elements might contribute to a higher proportion of cases (Liu et al., 2020). The conclusions of genome wide association studies (GWAS) are that most of the genetic risk for ASD resides in common genetic variants (Gaugler et al., 2014), but each individual common genetic variant exerts weak effects on ASD risk (Anney et al., 2012). Only two ASD GWAS loci have been replicated: chromosome 5p14.1 (Wang et al., 2009; Prandini et al., 2012; Wang et al., 2019) and chromosome 20p12.1 (Grove et al., 2019; Anney et al., 2010; Autism Spectrum Disorders Working Group of The Psychiatric Genomics Consortium, 2017). We published papers demonstrating that both of the replicated GWAS loci implicate lncRNAs as functional elements (DeWitt et al., 2016a; DeWitt et al., 2016b; Kerin et al., 2012; Liu et al., 2020). The chromosome 5p14.1 ASD GWAS signal implicates the lncRNA *MSNP1AS* (DeWitt et al., 2016a; DeWitt et al., 2016b; Kerin et al., 2012) and the chromosome 20p12.1 signal implicates the lncRNA *RPS10P2-AS1* (Liu et al., 2020). Both of these lncRNAs are significantly increased in expression in postmortem brains of individuals with ASD (Kerin et al., 2012; Liu et al., 2020). Overexpression of both *MSNP1AS* and *RPS10P2-AS1* in human neural progenitor cells caused a decrease in neurite complexity (DeWitt et al., 2016b; Liu et al., 2020).

ASD is a neurodevelopmental disorder that presents with altered social communication and behavioral flexibility. While the cause is not yet understood, research suggests that altered immunity contributes to ASD risk (Bottini et al., 2001; Braunschweig et al., 2008; Chen et al., 2013; Di Leva et al., 2024; Guan et al., 2019; Jash and Sharma, 2022; Krakowiak et al., 2017; Wang et al., 2023). Long noncoding RNAs (lncRNAs) are known to contribute to altered immune function and are also implicated in ASD etiology by genetic association (Jash and Sharma, 2022; Krakowiak et al., 2017). LncRNAs are genes that are transcribed yet not translated into a protein (Bertone et al., 2004; Ponting et al., 2009). The abundance of lncRNAs is directly correlated to the complexity of an organism, with the complex human brain expressing the greatest abundance of lncRNAs. In fact, lncRNAs are more abundant in the human brain than protein-coding RNAs (Derrien et al., 2012; Wan et al., 2016). The intent of this study is to determine if there is a convergence of altered immunity, ASD, and changes in expression of specific lncRNAs.

LncRNAs have recently become a target of interest in the study of ASD and other neurodevelopmental disorders (Aliperti et al., 2021; Ponting et al., 2009; Barry, 2014; Kinkade et al., 2024; Parikshak et al., 2016; Parikshak et al., 2013; Pourtavakoli et al., 2024; Sane et al., 2024; Turner et al., 2016; Iossifov et al., 2014). In a 2015 study, over 3,929 lncRNAs were differentially expressed in peripheral blood cells of individuals with ASD; 2,407 lncRNAs were upregulated while 1,522 lncRNAs were downregulated (Wang et al., 2015). Amongst the upregulated lncRNAs, 13 pathways were enriched in ASD. Amongst the downregulated lncRNAs, 14 pathways were enriched in ASD. Additionally, it was found the downregulated lncRNAs were associated with inflammation and immune response and were more likely to be involved in ASD. Comparatively, 2,610 protein coding mRNAs were differentially expressed with 1,789 upregulated while 821 were downregulated (Wang et al., 2015). Some studies have found there is significantly decreased expression of specific lncRNAs in children with ASD compared to controls (Sane et al., 2024) while other studies indicate that upregulation of specific lncRNA is associated with ASD (Parikshak et al., 2016; Parikshak et al., 2013). The latter studies found that amongst ASD subjects, there was increased expression of two specific lncRNAs found to interact with miRNA downregulated during neurotypical development yet upregulated in ASD (Parikshak et al., 2016; Parikshak et al., 2013; Wang et al., 2015; Jaffe et al., 2015). Consequently, it is understood that there is likely a relationship between lncRNAs and ASD, but the direction of this relationship requires additional research. Further literature indicates that lncRNAs play a role in immune function (Shu et al., 2025). This is of significance as individuals with ASD frequently present with comorbid altered immunity (Abdallah et al., 2012; Croen et al., 2005; Hodges et al., 2020; Masi et al., 2017). Specifically, an association has been found between ASD and Crohn’s disease, type I diabetes, and allergies (Croen et al., 2005; Xiang et al., 2018). Moreover, ASD is associated with increased levels of interleukins (Krakowiak et al., 2017).

Several publications identified lncRNAs that contribute to neurodevelopmental disorders (Alinejad-Rokny et al., 2020; Wang and Wang, 2020; Tong et al., 2020; Liu et al., 2020; Gudenas et al., 2017). All of these bioinformatics approaches provide strong statistical association, but do not attempt to determine functions of the lncRNAs in human neuronal cells. A brief outline of each of the publications:

1. Alinejad-Rokny et al. (2020) identified 266 lncRNAs in ASD-associated copy number variation (CNV) regions. The authors developed SNATCNV to identify 47 recurrent ASD CNV regions from 19,663 cases and 6,479 controls. They used FANTOM5 data to identify lncRNAs with brain-enriched patterns of expression. The authors conclude that there are 266 recurrently duplicated or deleted ASD candidate lncRNAs that are brain-enriched, with 94 of those lncRNAs dynamically expressed during iPSC neuronal differentiation. While the authors highlight one lncRNA (*LINC01238*) as the only gene–coding or noncoding–that is present within one of the CNV regions, they do not attempt any functional analysis of *LINC01238* (Alinejad-Rokny et al., 2020).
2. Wang and Wang (2020) identified 57 lncRNAs that were associated with ASD risk by 3 machine learning models. The authors used a machine learning approach to train models to predict protein-coding ASD risk genes based on developmental brain gene expression, then applied the models to predict lncRNAs associated with ASD. The 3 machine learning models predicted a total of 1,124 lncRNAs to be associated with ASD. There were 57 lncRNAs predicted to be associated with ASD by all three machine learning models (Wang and Wang, 2020). The authors provide a list of the top 5 lncRNA candidate genes, and include the well-studied *XIST* lncRNA, which is known to initiate X-chromosome inactivation and thus may be involved in sexually dimorphic processes (Wang and Wang, 2020).
3. Tong et al. (2020) identified 532 ASD-related lncRNAs based on genomic variant data of ASD from a twin study. The authors used gene ontology enrichment analysis of protein-coding genes in weighted gene co-expression networks analysis (WGCNA) modules with the 532 lncRNAs. These co-expression neighbors of ASD-related lncRNAs were enriched for processes such as nervous system development and transcriptional regulation. However, the authors provide no further functional analyses of the 532 lncRNAs.
4. Liu et al. (2020) compared GWAS for seven psychiatric traits (ASD, depression, bipolar disorder, schizophrenia, attention deficit hyperactivity disorder, anxiety and neuroticism) and identified 312 lncRNAs that were enriched across the psychiatric traits. The authors identified the lncRNA *LINC00461* as a gene with the highest pleiotropic effects across five psychiatric traits, and performed functional studies in mice that showed knockdown of the homologous lncRNA caused changes in behavior and delayed neuronal migration in the developing cerebral cortex (Liu et al., 2020). These data indicate that a lncRNA homologous to *LINC00461* can impact brain development in mice. However, the effect of *LINC00461* on human brain development has not been tested.
5. Gudenas et al. (2017) prioritized 126 candidate ASD-associated lncRNAs by integrating postmortem cerebral cortex RNA-seq and a developmental co-expression network. These data provide a rationally and data driven set of lncRNAs that may be involved in ASD, but none of the lncRNAs have a known function.

These well-designed hypothesis-generating studies identified >1,200 lncRNAs associated with ASD. These >1,200 ASD-associated lncRNAs are part of a much larger set of >30,000 lncRNAs expressed in the developing human brain (Hon et al., 2017). Each study provides a quantitative prioritization of the lncRNAs, but only one lncRNA is tested functionally; even then, the functionally tested lncRNA is a predicted mouse homolog of the lncRNA implicated in the human neurodevelopmental disorder, not the human lncRNA itself. Thus, none of these >1,200 neurodevelopmental disorder-relevant lncRNAs has been functionally tested in human neuronal cells (Table 1).

**TABLE 1:** The top 10 high-confidence lncRNAs.

LncRNA	Highlight
*LINC01933*	Tong et al. (2020): top ASD lncRNA
*HTR5A-AS1*	Gudenas et al. (2017): top ASD lncRNA
*LINC00461*	Liu et al. (2020): implicated in 7 neuropsychiatric conditions
*LINC00898*	Gudenas et al. (2017): top ASD lncRNA
*MSNP1AS*	Kerin et al. (2012): functional element of replicated ASD GWAS peak
*XIST*	Wang and Wang (2020): top ASD lncRNA
*LINC01546*	Gudenas et al. (2017): top ASD lncRNA
*LINC02607*	Wang and Wang (2020): top ASD lncRNA
*RPS10P2-AS1*	Bilinovich et al. (2019): functional element of ASD GWAS peak
*LINC01238*	Alinejad-Rokny et al. (2020): only gene in ASD-associated CNV

This study will review the literature and analyze publicly available databases to identify relationships among ASD, functional immune responses, and lncRNA, prevalence within both the brain and immune system. We expect that specific lncRNAs, play a role in the etiology of comorbid presentations of ASD, and immune dysregulation. The results from this study may provide insight regarding the biological markers and therapeutic targets for ASD, and altered immunity.

## Methods

To begin this project we first examined expression of ten previously identified long noncoding RNAs in various tissues including *LINC02607*, *LINC00461*, *HTR5A-AS1*, *RPS10P2-AS1*, *MSNP1AS*, *LINC01238*, *LINC01546*, *LINC01933*, *XIST*, and *LINC00898*.

We wished to understand if there was increased expression of the 10 lncRNAs discussed within the introduction (Table 1) in immune related tissues utilizing the online platform GTex. Further conduction of a literature analysis resulted in locating whole blood samples of males with ASD compared to controls collected by Kong et al. (2012). The AffyMetrix gene IDs that were left unpaired in relation to a gene symbol recorded in Kong et al. (2012) were identified and paired with the correct gene symbol. A further analysis was then conducted utilizing the online GTex portal to examine gene expression in tissues associated with immunity and reproduction. Subsequently, further analysis was conducted using Network Analyst to identify relationships in cell expressions and regulatory networks.

During the next phase of the study we identified a further meta-analysis conducted by Tylee et al. (2017) examining blood samples of individuals with and without autism (Tylee et al., 2017). We separated the genes that were upregulated in the sample compared to genes that were downregulated while maintaining a Covariate Controlled p value less than 0.05. We then utilized AI through the platform ChatGPT to identify lncRNAs within the set of genes and verified these results through additional literature analyses. Once we identified which lncRNAs were upregulated and downregulated in ASD patients compared to controls, we utilized Network Analyst to identify the signaling pathways, cells and transcription factors associated with each gene.

## Results

LncRNA *RSP102* had significant expression in EBV cultured lymphocytes, fibroblasts, and reproductive tissues (Figure 1a).

**FIGURE 1:**
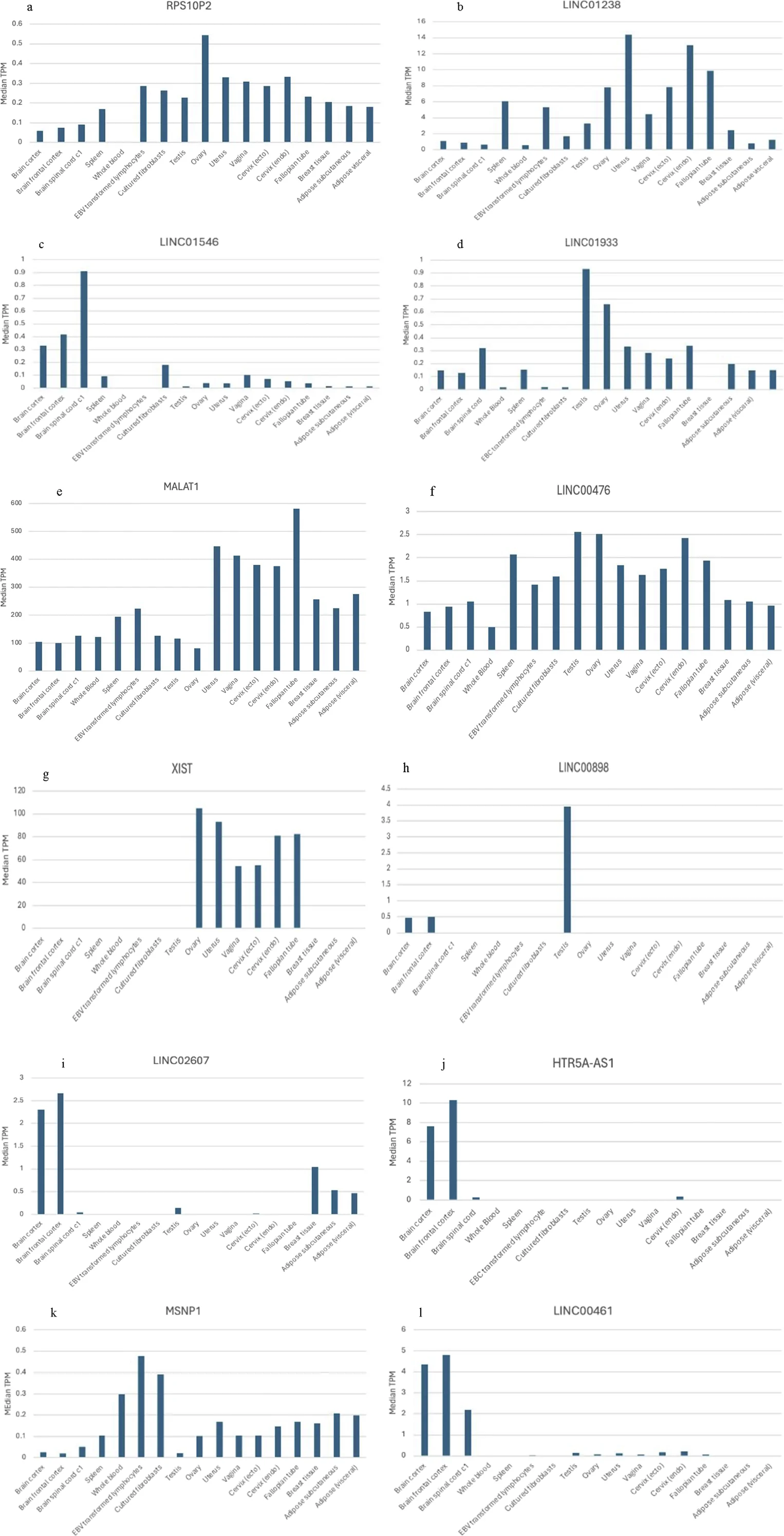
Tissue expression profile for the lncRNAs *RPS10P2, LINC01238, LINC01546, LINC01933, MALAT1, LINC00476, XIST, LINC00898, LINC02607, HTR5A-AS1, MSNP1, and LINC00461*.

*LINC01238* had significant expression in the spleen, cultured lymphocytes and reproductive tissues (Figure 1b). *LINC01546* (Figure 1c) had minimal expression in fibroblasts with the largest expression in the cortex and spinal cord and *LINC01933* (Figure 1d) had expression in male and female reproductive tissues.

Analyzing the data collected by Kong et al. (2012) we found the lncRNA *MALAT1* was highly expressed in the skin, ovary, uterus, vagina, and fallopian tube (Figure 1e). Results from GTex Portal and Network Analyst showed the lncRNA *LINC00476* was highly expressed in the brain, spleen, EBV lymphocytes, fibroblasts, and male and female reproductive tissues (Figure 1f). It was observed that the expression of LINC02607 (Figure 1i), LINC00461 (Figure 1l) and HTR5A-AS1 (Figure 1j) were significant in the adult cortex. MSNP1 (Figure 1k), LINC01238 (Figure 1b), LINC01546 (Figure 1c), and LINC01933 (Figure 1d) had expression in tissues associated with the immune system as well as pro-inflammatory cells. Additionally, RPS10P2, MSNP1, LINC01238, and LINC01933 had significant expression in sex specific tissues. *MSNP1* had significant expression in whole blood, lymphocytes and cultured fibroblasts (Figure 1k). XIST (Figure 1g), LINC00898 (Figure 1h), LINC02607 (Figure 1i).

Three lncRNAs associated with RNA binding were found via utilization of Network Analyst included *LOC220988, LINC01510* and *LINC02935* (Figures 2a,b).

**FIGURE 2:**
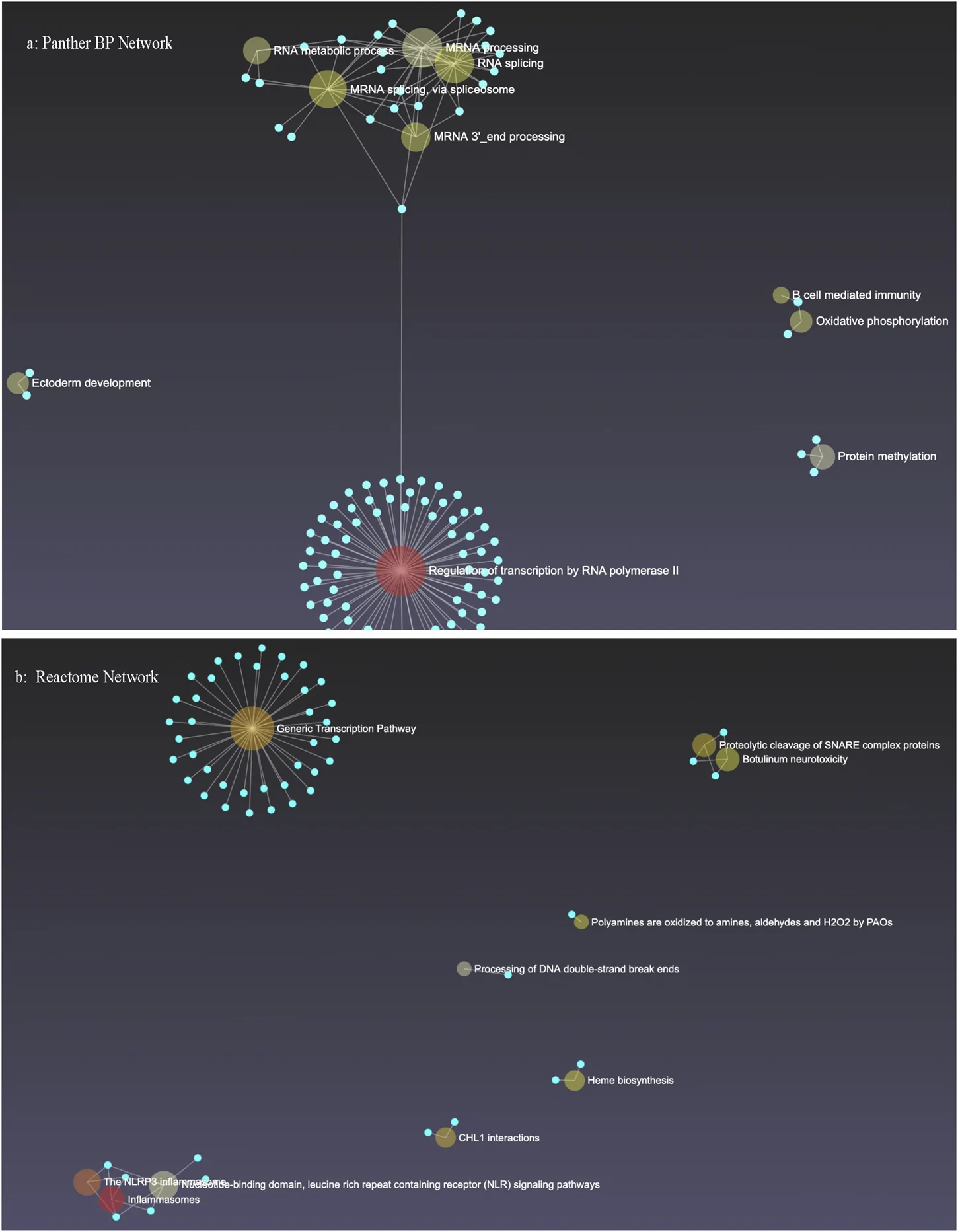
Reactome hub of transcription, with separate clusters for inflammasomes, DNA processing, heme biosynthesis, polyamine oxidation, and neurotoxicity pathways.

The third stage of the study examining the results from Tylee et al. (2017) demonstrated that of the 21,968 genes reported in the original dataset, 93 genes were lncRNAs that were significantly upregulated in people with ASD compared to controls (p < 0.05) (Table 2) (Tylee et al., 2017). In contrast, 51 genes were lncRNAs that were significantly downregulated in people with ASD compared to controls (p < 0.05) (Table 2).

**TABLE 2:** LncRNAs upregulated or downregulated in ASD patients (Tylee et al., 2017).

Upregulated	Downregulated
SNHG22	CDC42.IT1
MALAT1	OIP5.AS1
SNHG12	DAPK1.IT1
ZFAS1	PDCD4.AS1
SNHG7	SENCR
ZNF529.AS1	RARA.AS1
LINC00657	ENO1.IT1
MIR155HG	FAM13A.AS1
SNHG5	NUTM2A.AS1
SNHG6	ITPR1.AS1
EPB41L4A.AS1	RMST
OTUD6B.AS1	TLR8.AS1
SNHG8	LINC00202.1
LINC00323	LINC00963
LINC00216	TPT1.AS1
ST7.AS1	LOXL1.AS1
PRKCQ.AS1	LINC01137
LINC00493	MRVI1.AS1
LINC01254	RAD51.AS1
CASC18	LINC00685
PPP3CB.AS1	SND1.IT1
LINC00475	TMLHE.AS1
LINC01226	ZBED5.AS1
LINC00961	LINC01004
SNHG1	LINC01001
POT1.AS1	LINC00487
LINC00115	LINC01537
LRRC75A.AS1	LINC00877
LINC01521	JMJD1C.AS1
LINC00863	PCBP1.AS1
LINC01016	MIR22HG
MAPKAPK5.AS1	LINC01503
KRBOX1.AS1	FAM83A.AS1
LINC01532	MIR137HG
WDR11.AS1	NUTM2B.AS1
TOPORS.AS1	MIR600HG
ZMIZ1.AS1	FOXP1.IT1
PITPNA.AS1	LINC00665
ATP6V0E2.AS1	NR2F2.AS1
LDLRAD4.AS1	LINC01094
ANKRD10.IT1	LINC00565
LINC00680	MIR4435.1HG
LINC01359	FRY.AS1
JARID2.AS1	ENTPD3.AS1
ILF3.AS1	LINC00656
LINC00998	LINC00309
CHL1.AS1	CASC3
ZNF582.AS1	LINC00284
FOXD2.AS1	LINC01301
LINC00997	LINC00467
LINC00824	LINC01547
MIR133A1HG	​
LINC00427	​
LINC00299	​
RPARP.AS1	​
LINC01087	​
IQCH.AS1	​
LINC01158	​
SUCLG2.AS1	​
LINC00599	​
LINC00184	​
CASC1	​
HMGN3.AS1	​
LHX4.AS1	​
FOXN3.AS2	​
MYLK.AS1	​
TEX26.AS1	​
LINC00910	​
OSER1.AS1	​
LINC00086	​
LINC00092	​
LINC00938	​
LBX1.AS1	​
TMEM147.AS1	​
NCBP2.AS2	​
C9orf135.AS1	​
LINC00608	​
ARNTL2.AS1	​
LBX2.AS1	​
GNAS.AS1	​
NAPA.AS1	​
FAM181A.AS1	​
LINC01146	​
OVCH1.AS1	​
CASC23	​
LINC01169	​
CEBPA.AS1	​
KCNQ1OT1	​
LINC00528	​
SEC24B.AS1	​
LINC01420	​
NALCN.AS1	​
LINC00862	​

Among the 93 lncRNA genes that were upregulated, it was found the seed DNAI7 interacted with the transcription factors (TF) CEBPA, GATA6, FOXA1, TFAP2C, SOX2, FOXJ2, CTCF, and E2F1 (Figure 3). Further analysis using artificial intelligence platform Chat GPT showed that GATA6 interacted with *SNHG7*. FOXA1, TFAP2, and SOX2 interacted with the gene *MALAT1*. FOXJ2 interacted with the lnc RNA *LINC00657*. CTCF interacted with *MALAT1*, *SNHG1* and *KCNQ1OT1*. The transcription factor E2F1 interacted most strongly with *MALAT1*. We identified which genes were associated with CEBPA and found that the lncRNA *CEBPA.AS1*, *MALAT1, MIR155HG*, *SNHG5*, and *KCNQ1OT1* had significant interaction with CEBPA. Furthermore, we found that CEBPA plays a key role in immune response processes such as acute inflammatory response, myeloid leukocyte differentiation, leukocyte differentiation, cytokine mediated signaling pathways, hematopoietic or lymphatic organ development, negative regulation of cell proliferation, and positive regulation of cell differentiation (Table 3). CEBPA is also responsible for mitochondrial organization which suggests further research should be conducted regarding the role of CEBPA and maternal inheritance patterns. Lastly, it was observed CEBPA plays a key role in processes associated with ASD such as developmental maturation and sensory organ development. Further research will be required to identify amongst these pathways which are upregulated through *MALAT1* interaction with CEBPA and which are downregulated which will be the focus of future projects.

**FIGURE 3:**
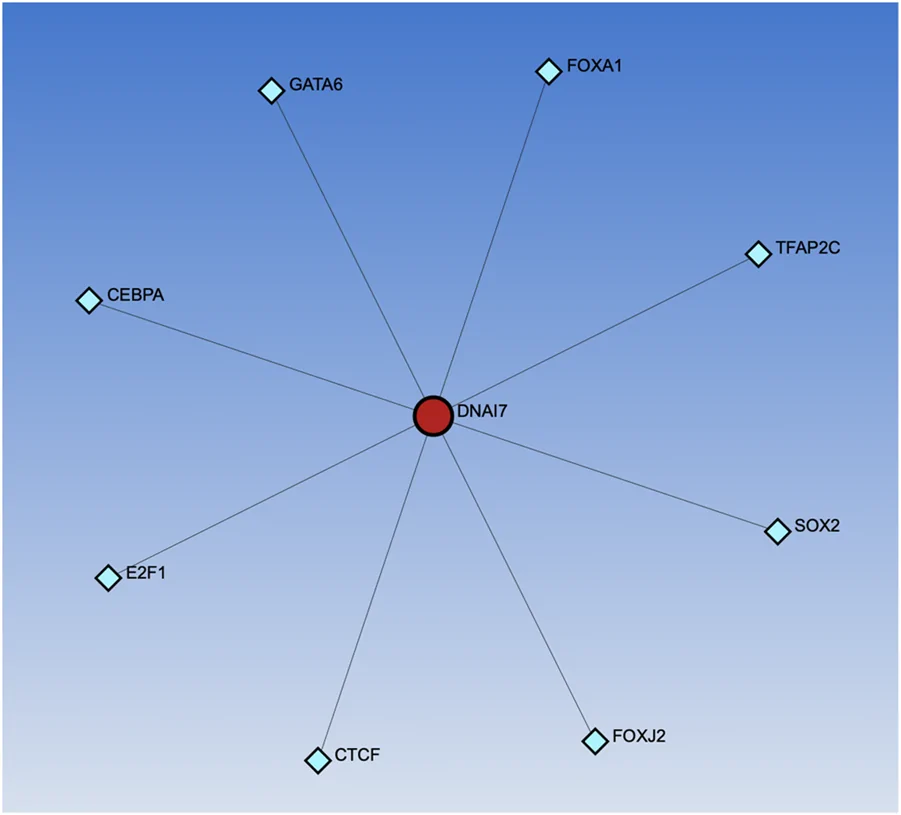
Upregulated lncRNA TF-gene interactions. Amongst the 51 lncRNAs that were downregulated in the dataset, we found interaction with the transcription factors TEAD2, CEBPA, CTCF, E2F4, TFAP2C, HIF1A, ETS1, HNF4A, SNAPC4, STAT1, STAT2, TEAD2, FOXC1, BRCA1, AR, E2F4, EOMES, SPI1, DACH1, E2F1, TP63, HMAP1, KLF9, HDGF, and MAZ.

**TABLE 3:** TEAD2 associated pathways and respective database (Included only the top five immune related pathways but will share further information upon request.).

*KEGG*	REACTOME	GO:BP	GO:MF	GO:CC	PANTHER:BP	PANTHER:MF
Acute myeloid leukemia	YAP1- and WWTR1 (TAZ)-stimulated gene expression	Acute inflammatory response	RNA polymerase II distal enhancer sequence-specific DNA binding transcription factor activity	Transcription factor complex	Cell-cell signaling	DNA_binding transcription factor activity
Non-alcoholic fatty liver disease (NAFLD)	Generic Transcription Pathway	Myeloid leukocyte differentiation	Sequence-specific DNA binding	Nucleoplasm	Regulation of gene expression, epigenetic	DNA_binding transcription factor activity, RNA polymerase II_specific
Hippo signaling pathway	Gene Expression	Cell maturation	DNA binding	Nuclear lumen	Cholesterol metabolic process	DNA binding
Transcriptional misregulation in cancer	​	Regulation of cell differentiation	Protein dimerization activity	Nuclear matrix	Mitochondrion organization	Protein binding
Pathways in cancer	​	Keratinocyte differentiation	Chromatin binding	Nucleus	Regulation of transcription by RNA polymerase II	​

Regarding the OmniPath network, significant interaction in Network Analyst was observed between *CASC 3* and the transcription factors TEAD2, CEBPA, CTCF, E2F4, TFAP2C, HIF1A, ETS1, HNF4A, SNAPC4, STAT1, and STAT2 (Figure 4a). Artificial intelligence analysis assessing interactions with downregulated lnc RNA showed interaction with TEAD2 and *MIR22HG* indirectly through LATS2/YAP regulation. Analysis with ChatGPT showed interaction with *MIR22HG* and CEBPA, CTCF, HIF1A, E2F4, and TFAP2C (Figure 4e). E2F4 also interacted with *LINC00665* while TFAP2C also interacted with *NCBP2.AS2*. STAT1 interacted with *OIP5-AS1* and *DLEU2* while STAT2 interacted indirectly with *OIP5-AS1*.

**FIGURE 4:**
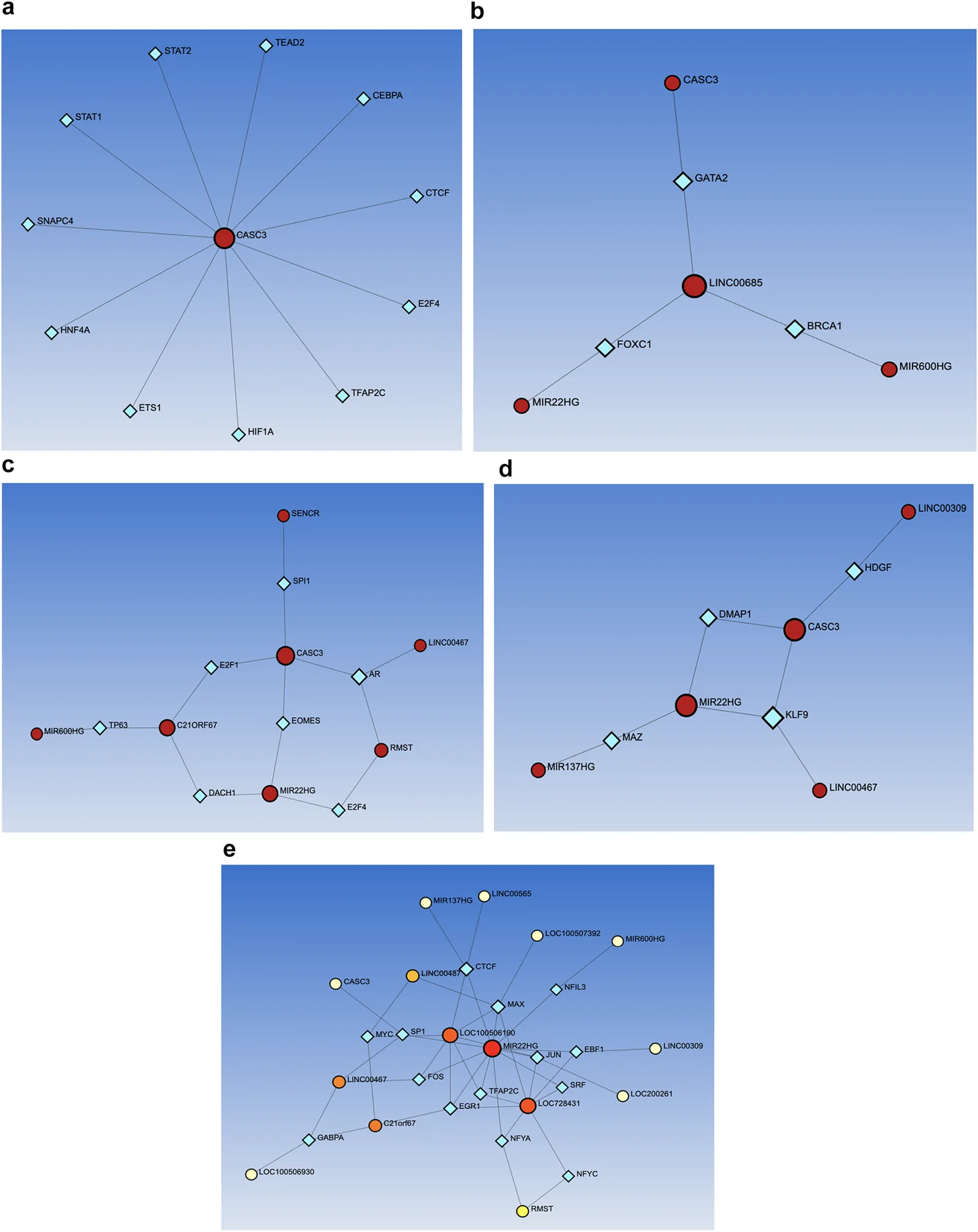
**(a)** Downregulated lncRNA TF-gene interactions OMNIPATH minimum network. **(b)** Downregulated lncRNA TF-gene interactions JASPAR minimum network. **(c)** Downregulated lncRNA TF-gene interaction ChEA Minimum Network. **(d)** Downregulated lnc RNA TF-gene interaction ENCODE Minimum Network. **(e)** Downregulated lnc RNA minimum network for TF-miRNA coregulatory network.

In the network JASPAR, significant interaction in Network Analyst was observed between the transcription factor GATA2 and the genes *CASC3* and *LINC00685*; the transcription factor BRCA1 and the genes *LINC00685* and *MIR600HG*; and the transcription factor FOXC1 with *MIR22HG* and *LINC00685* (Figure 4b). Artificial intelligence analysis with the platform ChatGPT showed interaction with *NR2F2-AS1* and the transcription factor FOXC1. Transcription factor BRCA1 was shown to also have interaction with *LINC0065*, *PDCD4-AS1*, *MIR22HG*, *OIP5-AS1*, *RMST*, and *LINC00467* where *LINC0065* was the strongest candidate. GATA2 had the strongest interaction with *CASC3*, *LINC00665* and *MIR22HG*. Within the ChEA network, significant interaction in Network Analyst was observed between the transcription factor TP63 and the genes *MIR600HG* and *C21ORF67* (Figure 4c). The transcription factor E2F1 had significant interaction with *CASC3* and *C21ORF67*. The transcription factor DACH1 had significant interaction with *C21ORF67* and *MIR22HG*. The transcription factor EOMES had significant interaction between the genes *MIR22HG* and *CASC3*. The transcription factor SPI1 had significant interaction between the genes *CASC3* and *SENCR*. The transcription factor AR had significant interaction between *CASC3*, *LINC00467* and *RMST*. The transcription factor E2F4 had significant interaction between *RMST* and *MIR22HG*. Analysis with ChatGPT showed TP63 interacts significantly with *LINC01503* and binding of the transcription factor to the gene locus activates transcription of the gene and such binding was found to be associated with squamous cell carcinoma. This analysis with ChatGPT showed the greatest candidates for interaction with the transcription factor E2F1 included *LINC001004* and *LINC01089*. The transcription factor SPI1 had an association with the lnc RNA’s *LINC01004, LINC00963,* and *MIR22HG*. *LINC01004* was found to be involved in SPI1 signaling in tumor associated macrophages, SPI1 was an enhancer of *LINC00963* expression, and lastly SPI1 and *MIR22HG* interaction were found to be presented in immune cell regulatory databases. Analysis with ChatGPT lastly demonstrated AR directly increases transcription of *LINC01503*, bind to regulatory regions near *MIR22HG*, and is expressed with *LINC00963* in transitioning from androgen to androgen independent prostate cancer.

Significant interaction in Network Analyst was observed between the transcription factor MAZ and the genes *MIR22HG* and *MIR137HG*; KLF9 and the genes *MIR22HG, LINC00467 and CASC3*; DMAP1 and the genes *CASC3* and *MIR22HG;* and HDGF and the genes *CASC3* and *LINC00309* (Figure 4d). Analysis with the AI platform ChatGPT showed MAZ may be involved with regulation of *CDC42-IT1*, *PDCD4-AS1*, *SENCR*, *TLR8-AS1*, *LINC00963, TPT1-AS1*, *RAD51-AS1*, *LINC00665*, *LINC00467*, *LINC00877*, *LINC01537*, *ENTPD3-AS1*, *LINC01547*, *MIR22HG*, *MIR600HG*, and *TMLHE-AS1*. This analysis also showed KLF9 is explicitly listed as a regulatory element for *LINC00877*, *ENTPD3-AS1*, *RAD51-AS1*, and *TLR8-AS1* and may be involved in regulation of *CDC42-IT1*, *PDCD4-AS1*, *SENCR*, *LINC00963*, *TPT1-AS1*, *LINC00665*, and *MIR22HG*.

## Discussion

This analysis highlighted the distinction between lnc RNA’s that are upregulated or downregulated in individuals with ASD compared to controls. Moreover, it highlighted the transcription factor networks correlated with these upregulated and downregulated lnc RNA’s. This work emphasized the processes associated with transcription factors which interact with downregulated lnc RNA’s in ASD. Transcription factors such as TEAD2 (Table 3), CEBPA (Table 4), CTCF (Table 5), E2F4 (Table 6), and TFAP2C (Table 7) are involved in acute inflammation. TFAP2C (Table 7) and HIF1A (Table 8) are involved in TGF-beta signaling. HIF1A (Table 8), ETS1 (Table 9) and HNF4A (Table 10) are involved in Th17 signaling. SNAPC4 (Table 11) is involved in gene transcription in several immune system cell types. STAT1 (Table 12), STAT2 (Table 13), and CEBPA (Table 4) are involved in cytokine signaling or binding. Th1 and Th2 signaling involves STAT1 (Table 12). In addition, transcription factors such as E2F4 (Table 6), TFAP2C (Table 7) and HIF1A (Table 8) are involved in TGF-beta signaling while Th17 signaling involves STAT1, HNF4A and HIF1A whereas Th1 and Th2 signaling involve STAT1. This highlights there is a correlation with interaction between transcriptome networks that interact with lnc RNA downregulated in ASD and transcriptome networks that are involved in immune response. Furthermore, eliciting that CEBPA interacts simultaneously with *MALAT1* which is upregulated in ASD and *MIR22HG* emphasizes there is a relationship that must be further investigated regarding the intersection between lnc RNA expression, immune dysregulation and ASD (Kita et al., 2018; Honarmand Tamizkar et al., 2021).

**TABLE 4:** CEBPA associated pathways and respective database (Included only the top five immune related pathways but there we will share further information upon request).

KEGG	GO:BP	GO:MF	GO:CC	PANTHER:BP	PANTHER:MF
Acute myeloid leukemia	Acute inflammatory response	RNA polymerase II distal enhancer sequence-specific DNA binding transcription factor activity	Transcription factor complex	Viral process	DNA-binding transcription factor activity
Non-alcoholic fatty liver disease (NAFLD)	Myeloid leukocyte differentiation	Transcription factor binding	Nuclear matrix	Cholesterol metabolic process	Protein binding
Transcriptional misregulation in cancer	Cytokine-mediated signaling pathway	Protein complex binding	Nucleoplasm part	Mitochondrion organization	DNA binding
Pathways in cancer	Leukocyte differentiation	Protein heterodimerization activity	Nucleoplasm	Transcription, DNA_templated	Chromatin binding
​	Inflammatory response	Protein homodimerization activity	Nuclear lumen	Transcription by RNA polymerase II	Nucleic acid binding

**TABLE 5:** CTCF associated pathways and respective database.

KEGG	GO:BP	GO:MF	GO:CC	PANTHER:BP	PANTHER:MF
Acute myeloid leukemia	Acute inflammatory response	RNA polymerase II distal enhancer sequence-specific DNA binding transcription factor activity	Transcription factor complex	Viral process	DNA-binding transcription factor activity
Non-alcoholic fatty liver disease (NAFLD)	Myeloid leukocyte differentiation	Sequence-specific DNA binding	Nuclear matrix	Regulation of gene expression, epigenetic	Protein binding
Transcriptional misregulation in cancer	Myeloid cell differentiation	Chromatin binding	Nucleoplasm	Cholesterol metabolic process	DNA binding
Pathways in cancer	Cell maturation	Protein complex binding	Nuclear lumen	Mitochondrion organization	Chromatin binding

**TABLE 6:** E2F4 associated pathways and respective database.

KEGG	Reactome	GO:BP	GO:MF	GO:CC	PANTHER:BP
Acute myeloid leukemia	Signaling by TGF-beta Receptor Complex	Acute inflammatory response	RNA polymerase II distal enhancer sequence-specific DNA binding transcription factor activity	Transcription factor complex	Blood circulation
TGF-beta signaling pathway	SMAD2/SMAD3:SMAD4 heterotrimer regulates transcription	Myeloid leukocyte differentiation	Transcription factor binding	Nucleoplasm	Regulation of gene expression, epigenetic
Cellular senescence	Transcriptional activity of SMAD2/SMAD3:SMAD4 heterotrimer	Transforming growth factor beta receptor signaling pathway	Protein domain specific binding	Nuclear lumen	Cholesterol metabolic process
Transcriptional misregulation in cancer	Generic Transcription Pathway	Cell maturation	Sequence-specific DNA binding	Nuclear part	Mitochondrion organization
Pathways in cancer	Cell Cycle	Regulation of cell differentiation	DNA binding	Nucleoplasm part	Regulation of cell cycle

**TABLE 7:** TFAP2C associated pathways and respective database.

KEGG	REACTOME	GO:BP	GO:MF	GO:CC	PANTHER:BP
TGF-beta signaling pathway	Signaling by TGF-beta Receptor Complex	Acute inflammatory response	Sequence_specific DNA binding	Transcription factor complex	Blood circulation
Cellular senescence	SMAD2/SMAD3:SMAD4 heterotrimer regulates transcription	Myeloid leukocyte differentiation	Protein dimerization activity	Nucleoplasm	Cell-cell signaling
Non-alcoholic fatty liver disease (NAFLD)	Transcriptional activity of SMAD2/SMAD3:SMAD4 heterotrimer	Cell maturation	Positive regulation of transcription, DNA_dependent	Nucleus	Regulation of gene expression, epigenetic
Transcriptional misregulation in cancer	Generic Transcription Pathway	Regulation of cell differentiation	Transcription from RNA polymerase II promoter	Nuclear part	Regulation of cell cycle
Pathways in cancer	Cell Cycle, Mitotic	Response to endogenous stimulus	DNA binding	Protein complex	Cell cycle

**TABLE 8:** HIF1A associated pathways and respective database.

KEGG	Reactome	GO:BP	GO:MF	GO:CC	PANTHER:BP
Th17 cell differentiation	Signaling by NOTCH	Positive regulation of cytokine production	Protein kinase binding	Transcription factor complex	Cell differentiation
TGF-beta signaling pathway	Signaling by TGF-beta Receptor Complex	Myeloid cell differentiation	Kinase binding	Nucleoplasm	Regulation of transcription
HIF-1 signaling pathway	Cellular response to hypoxia	Cytokine-mediated signaling pathway	Transcription factor binding	Cytosol	Regulation of catalytic activity
Autophagy - animal	Cellular responses to stress	Regulation of cytokine production	Sequence-specific DNA binding	Nucleolus	Negative regulation of apoptotic process
Mitophagy - animal	Regulation of Hypoxia-inducible Factor (HIF) by Oxygen	Inflammatory response	Protein dimerization activity	Nuclear part	Angiogenesis

**TABLE 9:** ETS1 associated pathways and respective database.

KEGG	GO:BP	GO:MF	GO:CC	PANTHER:BP
HTLV-I infection	Regulation of myeloid cell differentiation	Transcription factor binding	Transcription factor complex	Viral process
Cellular senescence	Myeloid cell differentiation	Sequence_specific DNA binding	Nucleoplasm part	Blood circulation
Renal cell carcinoma	Hemopoiesis	Positive regulation of transcription, DNA_dependent	Nucleoplasm	Regulation of transcription by RNA polymerase II
Ras signaling pathway	Hematopoietic or lymphoid organ development	Transcription from RNA polymerase II promoter	Protein complex	Regulation of gene expression, epigenetic
Pathways in cancer	Regulation of homeostatic process	DNA binding	Macromolecular complex	Regulation of catalytic activity

**TABLE 10:** HNF4A associated pathways and respective database.

KEGG	REACTOME	GO:BP	GO:MF	GO:CC	PANTHER:BP
Th17 cell differentiation	Nuclear Receptor transcription pathway	Transcription from RNA polymerase II promoter	Positive regulation of transcription, DNA-dependent	Nucleoplasm	Regulation of transcription by RNA polymerase II
JAK-STAT signaling pathway	Gene Expression	Response to carbohydrate stimulus	RNA polymerase II distal enhancer sequence-specific DNA binding transcription factor activity	Transcription factor complex	Regulation of gene expression, epigenetic
NOD-like receptor signaling pathway	Transcription	Regulation of hormone secretion	Steroid hormone receptor activity	Nuclear lumen	Blood circulation
Chemokine signaling pathway	RNA Polymerase III Transcription	Regulation of lipid metabolic process	Ligand-activated sequence-specific DNA binding RNA polymerase II transcription factor activity	Nuclear part	Viral process
Necroptosis	RNA Polymerase I, RNA Polymerase III, and Mitochondrial Transcription	Hormone secretion	Chromatin binding	Nucleoplasm part	Transcription by RNA polymerase II

**TABLE 11:** SNAPC4 associated pathways and respective database.

REACTOME	GO:BP	GO:MF	GO:CC	PANTHER:BP
RNA Polymerase III Transcription Initiation From Type 3 Promoter	Transcription from RNA polymerase II promoter	Chromatin binding	Transcription factor complex	Transcription by RNA polymerase II
RNA Polymerase III Transcription	RNA metabolic process	Transcription from RNA polymerase II promoter	Nucleoplasm part	Regulation of transcription by RNA polymerase II
RNA Polymerase I, RNA Polymerase III, and Mitochondrial Transcription	RNA biosynthetic process	DNA binding	Nucleoplasm	​
Transcription	Transcription, DNA-dependent	​	Nuclear lumen	​
Gene Expression	Cellular nitrogen compound biosynthetic process	​	Nuclear part	​

**TABLE 12:** STAT1 associated pathways and respective database.

KEGG	REACTOME	GO:BP	GO:MF	GO:CC	PANTHER:BP
Inflammatory bowel disease (IBD)	Interleukin-6 signaling	Defense response to virus	Cytokine receptor binding	Transcription factor complex	Blood circulation
Th1 and Th2 cell differentiation	Regulation of IFNG signaling	JAK_STAT cascade	Double_stranded DNA binding	Chromatin	Viral process
Toll-like receptor signaling pathway	Regulation of IFNA signaling	Regulation of I_kappaB kinase/NF_kappaB cascade	Structure_specific DNA binding	Nuclear chromatin	Transcription by RNA polymerase II
Th17 cell differentiation	Interferon alpha/beta signaling	I_kappaB kinase/NF_kappaB cascade	Sequence_specific DNA binding	Nuclear chromosome part	Regulation of transcription
NOD-like receptor signaling pathway	Interferon gamma signaling	Response to virus	DNA binding	Nuclear chromosome	​

**TABLE 13:** STAT2 associated pathways and respective database.

KEGG	REACTOME	GO:BP	GO:MF	GO:CC	PANTHER:BP
Osteoclast differentiation	Regulation of IFNA signaling	JAK-STAT cascade	Calcium ion binding	Nucleoplasm	Viral process
JAK-STAT signaling pathway	Interferon alpha/beta signaling	Defense response to virus	Transcription from RNA polymerase II promoter	Cytosol	Regulation of transcription by RNA polymerase II
NOD-like receptor signaling pathway	Interferon Signaling	Cytokine-mediated signaling pathway	DNA binding	Nuclear lumen	​
Chemokine signaling pathway	Cytokine Signaling in Immune system	Immune effector process	Cation binding	Nuclear part	​
Necroptosis	Immune System	Innate immune response	Ion binding	Organelle lumen	​

From the analysis conducted we observed the lncRNAs *DLEU2* and *OIP5-AS1* can induce the LPAATβ/PI3K/AKT/mTOR signaling pathway which is a signaling axis responsible for tumorigenesis and specifically has been found to drive cancer cell proliferation and apoptosis inhibition in osteosarcomas (Song et al., 2019). The lncRNA *RMST* is responsible for neuronal cell differentiation and interacts with *SOX2* to modulate neurogenesis (Tani, 2025; Ng et al., 2013) while *DLEU2* induces immune cell proliferation in kidney renal clear cell carcinoma and liver hepatocellular carcinoma (Fu et al., 2021; Klein et al., 2010). Moreover, the *DLEU2/miR-15a/16–1* cluster is responsible for controlling B cell expansion and deletion of the cluster results in mature B cell proliferation (Zhou et al., 2022). *LINC01094* is implicated in cancer cell proliferation through activation of the PI3K/AKT pathway and is responsible for cancer cell proliferation, metastasis, and immune cell infiltration (Luo et al., 2021). Overexpression of *LINC00963* is associated with lymphoid metastasis of gastric cancer with knockdown of *LINC00963* leading to enhanced apoptosis of gastric cancer cells (Zhu et al., 2020). *LINC00665* is positively associated with immune suppressive cytokines such as TGF-β, IL-10 and IL-1β and negatively associated with tumor suppressive cytokines such as IFN-γ, IL-2 and TNF-α (Han et al., 2024). Moreover, knockout of *LINC00665* leads to increased sensitivity of NK cells (Han et al., 2024). Downregulation of *MIR93* is associated with enhanced innate immune response through activation of the IFN/JAK/STAT pathway (Zhao et al., 2022; Guo et al., 2020). *MIR26B* can induce IFN expression and when overexpressed inhibits replication of non-segmented negative strand RNA viruses (Liu et al., 2018). However, *MIR26B* has recently become a target of interest with regard to its role in *Mycobacterium Tuberculosis* immune invasion as research shows upregulation of *MIR26B* leads to downregulation of NF-κB (Liu et al., 2018). *MIR137* is downregulated in peripheral blood samples of those with ASD and in mice *Mir137* upregulated could reduce autism like behaviors (Qin et al., 2025). Moreover, *MIR137* is associated with schizophrenia and is involved in emotional processing (Pergola et al., 2023). A study conducted by Tian et al. found that *MIR137* reduces apoptosis of astrocytes, inflammatory response and oxidative stress specifically within mouse models (Tian et al., 2020). This indicates further research should be conducted regarding the role of *MIR137* in inflammatory response, cytokine signaling and oxidative stress in human models. The results indicating *MIR137* is associated with a transcription factor network associated with immune cell differentiation poses future research questions regarding the role of *MIR137* in neural networks, particularly those surrounding emotional processing, and immune dysregulation.

An additional point of interest was the observation that a lncRNA downregulated in peripheral blood samples from individuals with autism spectrum disorder (ASD) was associated with the transcription factor GATA2. This finding is noteworthy, as GATA2 plays a critical role in neuronal development and differentiation (Bellomi et al., 2025). Furthermore, GATA2 deficiency has been linked to immunodeficiency, characterized by the loss of multiple immune cell populations, including B cells, dendritic cells, and natural killer cells (Fabozzi et al., 2022; National Institute of Allergy and Infectious Diseases; Nováková et al., 2016).

Future studies will aim to elucidate the relationship between GATA2 and the downregulated lncRNA identified in peripheral blood samples from individuals with ASD. In addition, further investigation is warranted to clarify the role of GATA2 in immune dysregulation, as well as to determine how lncRNAs that interact with GATA2 may influence immune responses and neural development.

Future studies will focus on further elucidating the role of specific lncRNAs in ASD and immune dysregulation. Induced pluripotent stem cells (iPSCs) with either upregulated or downregulated lncRNA expression will be generated and differentiated into cerebral organoids to model neurodevelopmental processes. In parallel, hematopoietic stem cell assays will be employed to derive immune cell lineages, enabling investigation of the effects of these lncRNA on immune cell proliferation and differentiation. This integrated approach will allow for systematic evaluation of the intersection between lncRNA function, immune system regulation, and ASD pathophysiology, with particular emphasis on defining the mechanistic role of lncRNAs in immune dysregulation.

## Data Availability

The original contributions presented in the study are included in the article/supplementary material, further inquiries can be directed to the corresponding author.
